# The Safety and Efficacy of Platelet-Rich Plasma in Enhancing Outcomes Following Circumcision in Children

**DOI:** 10.3390/jcm14217620

**Published:** 2025-10-27

**Authors:** Tahsin Onat Kamci, Mustafa Azizoglu, Sergey Klyuev, Mehmet Hanifi Okur, Hakkari Aydogdu, Maria Escolino, Asli Pinar Zorba Yildiz, Ciro Esposito, Sameh Shehata

**Affiliations:** 1 Department of Pediatric Surgery, Tatvan State Hospital, 13200 Bitlis, Turkey; 2 Pediatric Surgery Stem Cell Association Study Group (PESSCA), 34510 Istanbul, Turkey; 3 Department of Pediatric Surgery and Pediatric Urology, Dicle University, 21830 Diyarbakir, Turkey; 4 Department of Pediatric Surgery and Pediatric Urology, Istanbul Esenyurt Necmi Kadioglu State Hospital, 34510 Istanbul, Turkey; 5 Department of Stem Cell and Tissue Engineering, Faculty of Health Sciences, Istinye University, 34010 Istanbul, Turkey; 6 Department of Pediatric Surgery, AO GK MEDSI, 102151 Moscow, Russia; 7 Division of Pediatric Surgery, Federico II University Hospital, 80131 Naples, Italy; 8 Vocational School of Health Care Services, Istinye University, 34010 Istanbul, Turkey; 9 Pediatric Surgery, Alexandria University, Alexandria 22301, Egypt

**Keywords:** platelet-rich plasma, circumcision, penile edema, wound healing

## Abstract

**Background**: The primary objectives of platelet-rich plasma (PRP) therapy are to enhance the wound-healing process, reduce pain, and minimize the loss of productivity due to recovery time. Localized application of PRP, which is enriched with growth factors such as PDGF, TGF-β1, IGF-1, VEGF, and FGF-2, as well as interleukins (IL-1, IL-4, IL-6, IL-10, and IL-13), has been documented to accelerate the healing process by approximately 30–40%. This study aimed to assess the safety and efficacy of platelet-rich plasma (PRP) in enhancing outcomes following circumcision in male children. **Methods**: The patients were divided into two groups: one undergoing standard circumcision and the other receiving PRP application during circumcision. Pain scores, edema level, bleeding, local infection, and safety of PRP were evaluated. **Results**: This study evaluated 80 male children undergoing circumcision, divided into two groups: Group CS (n = 44) underwent classical circumcision, and Group PRP (n = 36) received PRP application. Median ages were comparable (*p* = 0.101). Penile edema occurred less frequently in the PRP group (5.6%) compared to the CS group (18.2%) (*p* = 0.089), with no severe edema observed in the PRP group. Postoperative bleeding was present in 6.8% of the CS group but absent in the PRP group (*p* = 0.110). Other complications, such as nausea (CS: 6.8%, PRP: 5.6%, *p* = 0.816), vomiting (CS: 4.5%, PRP: 2.8%, *p* = 0.679), local infection (CS: 2.3%, PRP: 0%, *p* = 0.363), wound dehiscence (CS: 2.3%, PRP: 0%, *p* = 0.363), and skin tunnel formation (CS: 6.8%, PRP: 2.8%, *p* = 0.409), showed no significant differences. No cases of necrosis, chordee, rotational anomaly, or secondary phimosis were observed. Safety analysis of PRP revealed minor complications during blood draw: hypotension in one patient (2.8%) and local ecchymosis in two patients (5.6%), resolving without intervention. During PRP application, one allergic reaction (2.8%) occurred, presenting as a transient rash that resolved spontaneously. Group PRP consistently reported lower pain scores than Group CS at all time points. **Conclusions**: PRP application during circumcision is safe. The findings provide preliminary but important evidence regarding the potential benefits of PRP in pediatric circumcision.

## 1. Introduction

Male circumcision, a surgical procedure involving the removal of the foreskin from the penis, has been performed for thousands of years, mainly for religious, cultural, or medical purposes [1,2]. Over the years, circumcision techniques have advanced, with a focus on reducing pain, minimizing complications, improving cosmetic results, and expediting recovery [1,2,3].

Although circumcision is one of the most commonly performed surgical procedures and has a low complication rate, it is not entirely risk-free. Even minor issues such as penile edema can be enough to cause significant concern and panic for the patient’s parents. Circumcision complications can be divided into minor and major complications. Minor complications that may arise shortly after circumcision include bleeding, improper skin removal, and infection at the surgical site, all of which are relatively frequent. More serious early complications, although less common, can involve glandular amputation, urinary retention, urethral damage, and penile tissue death. Long-term complications, occurring later, encompass issues such as adhesions, formation of skin bridges, epidermal cysts, phimosis, curvature of the penis (chordee), entrapment of the penis, urethrocutaneous fistulas, and narrowing of the urethral opening (meatal stenosis) [4,5,6]. Among the complications associated with traditional circumcision, edema is the most prevalent, reported in 16% to 55% of cases, followed by bleeding at 2.9% [6,7,8]. Infection ranks as the third most frequent issue, occurring in 2.5% of cases [7,8].

The primary objectives of platelet-rich plasma (PRP) therapy are to enhance the wound-healing process, reduce pain, and minimize the loss of productivity due to recovery time [9,10]. Localized application of PRP, which is enriched with growth factors such as PDGF, TGF-β1, IGF-1, VEGF, and FGF-2, as well as interleukins (IL-1, IL-4, IL-6, IL-10, and IL-13), has been documented to accelerate the healing process by approximately 30–40% [11,12,13,14,15,16]. TGF-β plays a crucial role in regulating inflammatory responses and enhancing collagen synthesis, facilitating the repair of damaged tissues [13]. It also helps minimize fibrosis while improving the elasticity and functional integrity of penile tissue [13]. VEGF stimulates endothelial cell proliferation and promotes angiogenesis, thereby improving blood flow to the penile tissue [14,15].

This study aimed to assess the safety and efficacy of PRP in enhancing outcomes following circumcision in children and explore the safety, efficacy, and feasibility of applying PRP in pediatric penile surgeries as a preliminary investigation.

## 2. Materials and Methods

### 2.1. Patients and Groups

**Figure 1:**
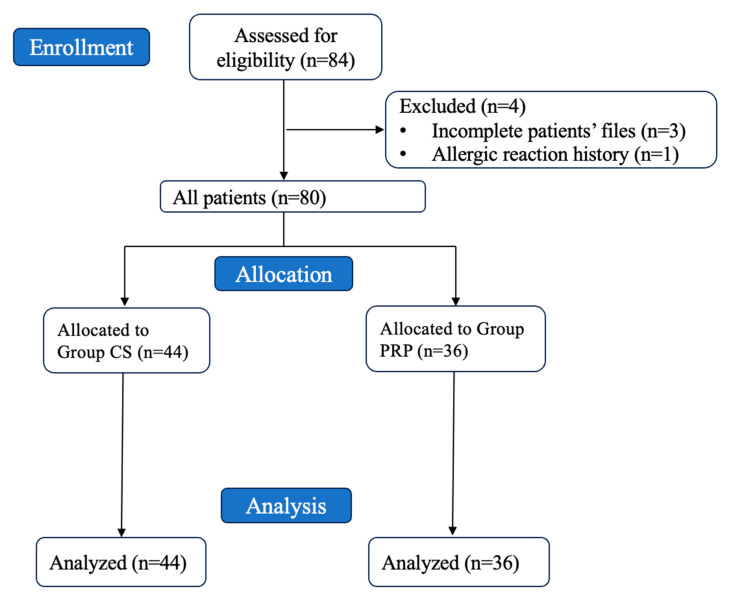
Flow chart of patient selection.

This retrospective cohort study aimed to evaluate the safety and efficacy of PRP in wound healing in children undergoing circumcision. The study population included 80 male children aged 6–18 years who underwent circumcision at Istanbul Esenyurt Necmi Kadioglu Hospital and Dicle University between 20 October 2023 and 28 February 2024. The patients were divided into two groups: Group CS consisted of children who underwent circumcision alone, while Group PRP included children who received an intraoperative PRP injection at the surgical site during the circumcision procedure. The patient selection flow chart is given in Figure 1.

Data were retrospectively collected from hospital records, including demographic information such as age, medical history, and comorbidities, as well as clinical outcomes such as the incidence of wound infection, the presence and duration of edema, the need for reoperation, time to complete wound healing, and cosmetic outcomes as assessed by parents and clinicians.

### 2.2. Eligibility Criteria

Patients with known coagulopathies, allergic reaction history, having allergic reactions to blood transfusions or any medications, hematologic disorders, pre-existing infections, systemic conditions affecting wound healing, or any other comorbidity, and incomplete medical records were excluded from the study.

### 2.3. PICO Strategy

*Population(s):* Male children underwent circumcision aged between 6 and 18 years.

*Intervention:* Patients underwent PRP administration in the same session as circumcision.

*Comparison:* Patients underwent classical circumcision without PRP administration.

*Outcome(s):* Evaluation of safety (complications such as edema, bleeding, and infection) and efficacy (pain reduction and reduced postoperative complications).

### 2.4. Preparation of PRP

**Figure 2:**
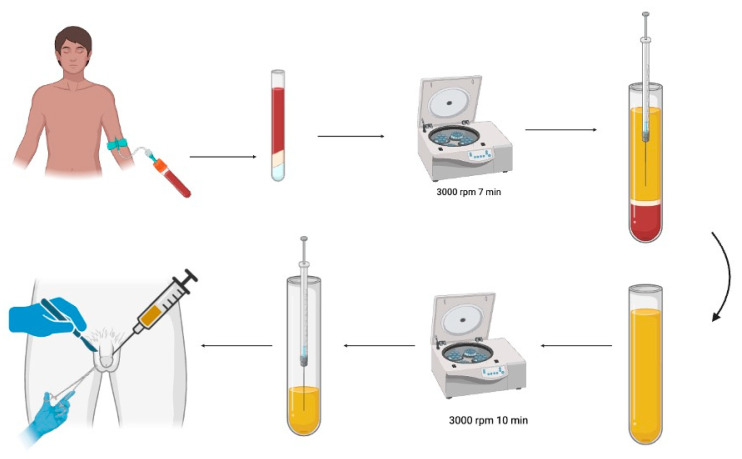
PRP preparation and application model.

PRP is widely used in clinical applications, with its production adhering to good manufacturing practices. The standard PRP production method involves a two-step centrifugation process. PRP is prepared using a previously described technique [16]. Initially, erythrocyte concentrate and platelet-containing plasma are separated by the first centrifugation. A subsequent centrifugation step isolates the platelet-poor plasma (PPP) and PRP/platelet concentrate. PRP was prepared using a standardized centrifugation protocol to obtain a concentrated solution rich in growth factors. Autologous blood was collected from the patients and processed to isolate PRP, which was immediately injected into the wound site under sterile conditions following circumcision (Figure 2).

### 2.5. Application Technique of PRP

**Figure 3:**
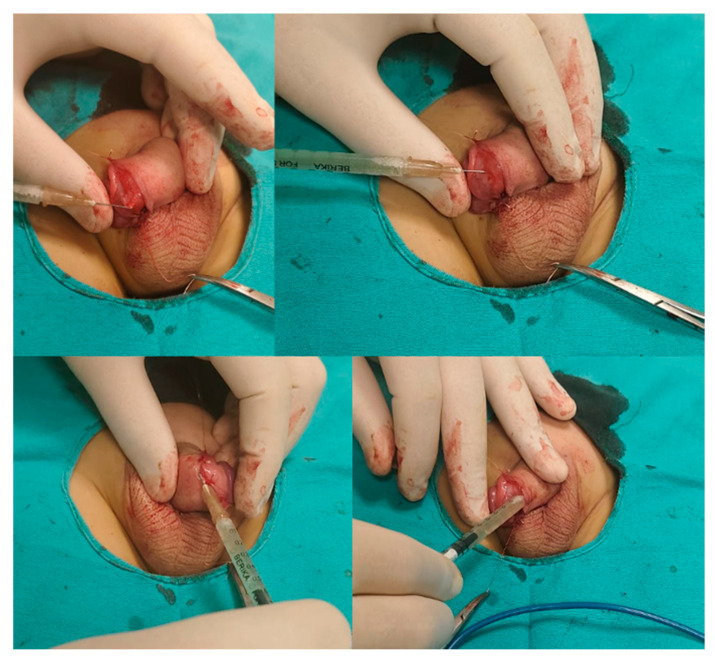
Application technique of PRP.

Group CS patients received no additional treatment beyond the standard circumcision procedure. In Group PRP, each patient underwent circumcision, and after the skin and mucosal incision, but before suturing, 0.5 mL of PRP was injected into the wound edges (with a 26 gauge needle) and subcutaneous tissue. Additionally, 0.5 mL of PRP was dripped onto the tissue surface. The procedure was completed with repair using 5/0 Vicryl sutures (Figure 3).

### 2.6. Potential PRP Mechanism of Action

**Figure 4:**
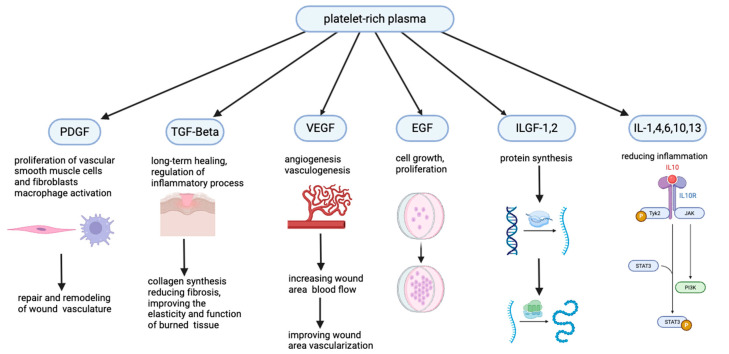
Potential mechanism and effects of PRP.

PRP is rich in coagulation factors, growth factors (PDGF, TGF-β1, IGF-1, VEGF, and FGF-2), and interleukins (IL-1, IL-4, IL-6, IL-10, and IL-13), which collectively promote tissue repair by stimulating cell proliferation, decreasing cytokine release, and reducing cell apoptosis [9,10,11,12]. These factors also attract stem cells, restore metabolic activity, and mitigate oxidative stress, further enhancing wound healing [10,11]. Additionally, PRP’s biological mechanisms include anti-inflammatory effects, such as promoting monocyte differentiation into anti-inflammatory phenotypes, reducing pro-inflammatory M1 macrophages, and activating dendritic cells. This combination of processes underscores the therapeutic potential of PRP in clinical practice, effectively reducing tissue necrosis and accelerating recovery [9,10,11,12] (Figure 4).

### 2.7. Sample Size Calculation

A priori sample size estimation was conducted using a two-sample *t*-test (two-sided, α = 0.05). The calculation was based on an expected reduction in VAS scores from 3.4 ± 1.5 to 2.1 ± 1.2, corresponding to a large effect size (Cohen’s d = 0.96). For 95% statistical power, a minimum of 29 patients per group (total 58) was required. To account for a potential dropout rate of 10%, the final target enrollment was set at 32 patients per group, yielding a total of 64 participants.

### 2.8. Statistical Analysis

Statistical analysis was performed using SPSS v26.0 and JAMOVI software v2.4.1. The normality of data distribution was tested using the Shapiro–Wilk test. As the data in our study did not follow a normal distribution, non-parametric tests (Mann–Whitney U) were used for the analysis. In our study, categorical data were analyzed using the chi-square test. A *p*-value of less than 0.05 was considered statistically significant.

## 3. Results

This study included 80 male children who underwent circumcision, divided into two groups: Group CS (n = 44) underwent classical circumcision, while Group PRP (n = 36) received PRP application during the procedure. The median age was comparable between the groups (CS: 11 years; PRP: 10 years, *p* = 0.101). Penile edema occurred less frequently in the PRP group (5.6%) compared to the CS group (18.2%), with a trend toward statistical significance (*p* = 0.089). Among those with edema, the severity differed: in Group CS, 50% of cases were categorized as severe, while no severe edema was observed in the PRP group. Postoperative bleeding occurred in 6.8% of the CS group but was entirely absent in the PRP group (*p* = 0.110). Other complications, including nausea (CS: 6.8%, PRP: 5.6%, *p* = 0.816), vomiting (CS: 4.5%, PRP: 2.8%, *p* = 0.679), local infection (CS: 2.3%, PRP: 0%, *p* = 0.363), wound dehiscence (CS: 2.3%, PRP: 0%, *p* = 0.363), and skin tunnel formation (CS: 6.8%, PRP: 2.8%, *p* = 0.409), did not show statistically significant differences between groups. There were no occurrences of necrosis, chordee, rotational anomaly, meatal stenosis, or secondary phimosis in either group (*p* > 0.05 for each comparison) (Table 1).

Complications during blood draw and PRP application were minimal, reflecting the overall safety of the procedures. Among the 36 patients in the PRP group, complications during blood draw included hypotension in 1 patient (2.8%) and local ecchymosis in 2 patients (5.6%). No allergic reactions were observed during the blood draw process. The patient who developed hypotension experienced it due to anxiety. No additional intervention was required; the patient was placed in a supine position on a patient bed, and their blood pressure normalized within a few minutes. For the two patients who developed local ecchymosis, simple pressure was applied to the affected area. After five days, no significant ecchymotic marks were observed in either patient. During the application of PRP, no instances of hypotension or local ecchymosis were reported during PRP application. One allergic reaction was observed in the PRP group 5 min after application. The patient who experienced an allergic reaction developed a few rash-like lesions on his body, which resolved spontaneously within minutes without requiring any medical intervention. The patient was placed under close observation, including regular blood pressure monitoring. Since no complications arose throughout the day, the patient was safely discharged (Table 2).

**Figure 5:**
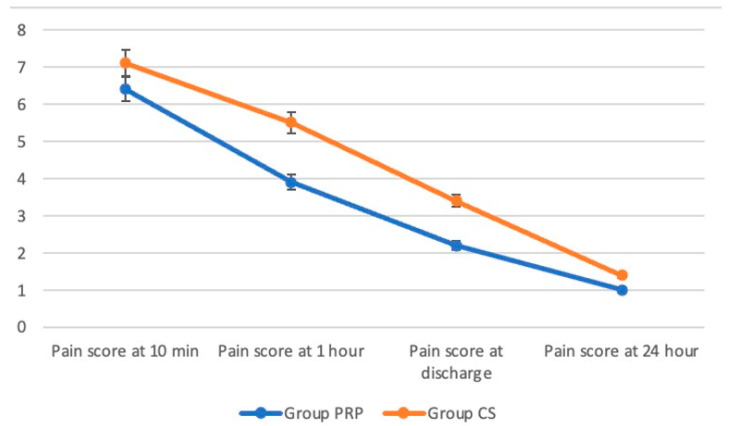
VAS scores.

Figure 5 illustrates the Visual Analogue Scale (VAS) pain scores at various time points (10 min, 1 h, discharge, and 24 h) for Group PRP and Group CS. Group PRP consistently reported lower pain scores than Group CS at all time points (Figure 5).

## 4. Discussion

The study demonstrated that PRP application during circumcision is safe. PRP application was associated with lower rates of penile edema and postoperative bleeding, as well as consistently lower pain scores, although most differences did not reach statistical significance. These findings suggest a possible beneficial effect that warrants confirmation in larger, prospective studies. The procedure was well-tolerated, with minimal complications such as transient hypotension and ecchymosis during blood draw. An allergic reaction occurred in one PRP patient, resolving spontaneously. These findings highlight the potential of PRP as a safe and effective adjunct in pediatric circumcision, enhancing recovery and minimizing postoperative complications.

In studies involving adults, particularly for erectile dysfunction (ED), the application of PRP is observed to be quite common. Poulios et al.’s placebo-controlled experiment in 2021 demonstrated superior outcomes in the PRP group compared to the placebo group [17]. Similarly, a meta-analysis by Du et al. concluded that PRP, either alone or in combination with ESWT, is a safe and effective treatment option for ED patients [15]. In contrast, PRP applications related to penile surgeries in children are extremely rare and almost nonexistent. To the best of our knowledge, this study is the first to analyze the complications of PRP following circumcision in children and to test its safety in pediatric penile surgery.

PRP enhances wound healing by delivering concentrated growth factors such as PDGF, TGF-β1, IGF-1, VEGF, and interleukins [18,19]. These factors stimulate cell proliferation, promote angiogenesis, reduce inflammation, and accelerate tissue repair [18,19,20]. PRP minimizes fibrosis, restores tissue elasticity, and reduces oxidative stress, making it a promising adjunct for surgical recovery [13,15]. Another primary reason for conducting this study on circumcision patients was to design a preliminary framework for future PRP applications in hypospadias surgeries and to evaluate its safety and efficacy in pediatric penile surgeries, similar to its established use in adults. Indeed, PRP could play a significant role as a wound-healing agent in hypospadias and other penile surgeries. Our study results demonstrate that PRP is both effective and safe when used in circumcision.

Limitations

Our study has several limitations. First, the sample size was relatively small, limiting the generalizability of the findings. Second, the short follow-up period prevented the evaluation of long-term outcomes and complications. Third, the study was conducted in a single center, possibly introducing selection bias. Fourth, the subjective nature of pain scoring could have led to variability in reported outcomes. Lastly, while PRP preparation and application followed standardized protocols, variations in PRP quality and concentration across different settings were not explored, which could influence the reproducibility of the results. Future studies with larger, multicenter cohorts and longer follow-up durations are recommended. Although this study includes a comparative analysis between PRP-assisted and classical circumcision, it also has the nature of a case series. The comparative results should therefore be interpreted cautiously, as the methodology has inherent limitations. Nevertheless, presenting the two groups side by side provides useful preliminary insights and may serve as a basis for future prospective studies.

Future directions

Given its biological properties, PRP may have potential applications beyond circumcision, particularly in hypospadias surgery. PRP is rich in growth factors that enhance neovascularization and tissue regeneration, which could theoretically improve flap vascularization and reduce ischemia-related complications. By promoting better tissue perfusion and wound healing, PRP may lower the risk of fistula formation and urethral stricture, two of the most common challenges in hypospadias repair. Future well-designed multicenter randomized trials are warranted to investigate whether these potential benefits can be translated into clinical practice in pediatric urology.

## 5. Conclusions

PRP application during circumcision is safe. This study has certain limitations. As a single-center retrospective study with a relatively small cohort, the conclusions cannot be generalized to all populations. Nonetheless, the findings provide preliminary but important evidence regarding the potential benefits of PRP in pediatric circumcision. Future multicenter studies with larger sample sizes and prospective designs are warranted to strengthen the evidence base and confirm these results.

## Figures and Tables

**Table 1:** Comparison of treatment groups.

	Group CS (n = 44)	Group PRP (n = 36)	*p*-Value
Age	11 (9; 13.3)	10 (9; 12.4)	0.101
Nausea	3 (6.8%)	2 (5.6%)	0.816
Vomiting	2 (4.5%)	1 (2.8%)	0.679
Penile edema	8 (18.2%)	2 (5.6%)	0.089
Edema degree			0.335
Mild	1 (12.5%)	1 (50%)	
Moderate	3 (37.5%)	1 (50%)	
Severe	4 (50%)	0 (0%)	
Postoperative bleeding	3 (6.8%)	0 (0%)	0.110
Necrosis	0 (0%)	0 (0%)	1.000
Local infection	1 (2.3%)	0 (0%)	0.363
Wound dehiscence	1 (2.3%)	0 (0%)	0.363
Skin tunnel	3 (6.8%)	1 (2.8%)	0.409
Chordee after procedure	0 (0%)	0 (0%)	1.000
Rotational anomaly after procedure	0 (0%)	0 (0%)	1.000
Meatal stenosis	0 (0%)	0 (0%)	1.000
Secondary phimosis	0 (0%)	0 (0%)	1.000

PRP: platelet-rich plasma; CS: classical circumcision.

**Table 2:** Safety analysis.

	n (%)
Complications during blood draw	
Hypotension	1 (2.8%)
Allergic reaction	0 (0%)
Local ecchymosis	2 (5.6%)
Complications during PRP application	
Hypotension	0 (0%)
Allergic reaction	1 (2.8%)
Local ecchymosis	0 (0%)

PRP: platelet-rich plasma.

## Data Availability

The datasets generated during and/or analyzed during the current study are available from the corresponding author on reasonable request.
